# The Abcc6a Knockout Zebrafish Model as a Novel Tool for Drug Screening for Pseudoxanthoma Elasticum

**DOI:** 10.3389/fphar.2022.822143

**Published:** 2022-03-04

**Authors:** M. Van Gils, A. Willaert, P. J. Coucke, O. M. Vanakker

**Affiliations:** ^1^ Center for Medical Genetics, Ghent University Hospital, Ghent, Belgium; ^2^ Department of Biomolecular Medicine, Ghent University, Ghent, Belgium

**Keywords:** pseudoxanthoma elasticum, zebrafish, compound screening, alendronate, etidronate, magnesium citrate, sodium thiosulfate, vitamin K1

## Abstract

Pseudoxanthoma elasticum (PXE) is a multisystem ectopic mineralization disorder caused by pathogenic variants in the ABCC6 gene. Though complications of the disease can be treated, PXE itself remains currently intractable. A strategy for rapid and cost-effective discovery of therapeutic drugs would be to perform chemical compound screening using zebrafish, but this approach remains to be validated for PXE. In this paper, we validate a stable CRISPR/Cas9 abcc6a knockout zebrafish model–which has spinal column hypermineralization as its primary phenotypic feature–as a model system for compound screening in ectopic mineralization. We evaluated the anti-mineralization potential of five compounds, which had (anecdotal) positive effects reported in Abcc6 knockout mice and/or PXE patients. Abcc6a knockout zebrafish larvae were treated from 3 to 10 days post-fertilization with vitamin K1, sodium thiosulfate, etidronate, alendronate or magnesium citrate and compared to matching controls. Following alizarin red S staining, alterations in notochord sheath mineralization were semiquantified and found to largely congrue with the originally reported outcomes. Our results demonstrate that the use of this abcc6a knockout zebrafish model is a validated and promising strategy for drug discovery against ectopic mineralization.

## 1 Introduction

Pseudoxanthoma elasticum (PXE) is an ectopic calcification disorder caused by biallelic mutations in the ABCC6 (ATP-Binding Cassette, subfamily C member 6)–and in rare cases the ENPP1 (Ectonucleotide Pyrophosphatase/Phosphodiesterase 1)–gene (Nitschke et al., 2012; Nitschke and Rutsch 2012). In PXE aberrant hydroxyapatite crystals are progressively deposited onto elastic fibers, which consequently lose their elastic properties and fragment, leading to a myriad of symptoms. Generally, patients develop skin lesions (papular plaque formation and excessive skin folds in flexural regions), ocular symptoms (peau d’orange, angioid streaks, choroidal neovascularizations, retinal haemorrhaging and vision loss) and cardiovascular manifestations (e.g., peripheral artery disease, increased risk of stroke). The phenotypic manifestations of PXE show a high degree of inter- and intrafamilial variability without solid genotype-phenotype correlations, making it difficult for health care professionals to accurately manage patients. Moreover, existing treatment options for PXE are limited and currently focus on slowing down disease progression in patients through lifestyle changes or by treating complications via e.g., cosmetic surgery or intravitreal injection of anti-VEGF antibodies (Vascular Endothelial Growth Factor; Shimada et al., 2021).

As such, there is a pressing need for therapeutic interventions in patient care and in recent years several compounds have been tested for their potential to halt PXE. Some compounds have been described casuistically, such as sodium thiosulfate (STS) (Omarjee et al., 2020). Others, such as vitamin K1 (VK1), have been tested in PXE knockout mice and recently in two PXE zebrafish models with mixed results. In zebrafish models VK1 significantly reduced mineralization (Mackay et al., 2015; Sun et al., 2021) but it did not affect mineralization in murine models (Brampton et al., 2011; Gorgels et al., 2011; Jiang et al., 2011). Similarly, treatment with bisphosphonates showed that etidronate (etid.), but not alendronate (alen.), could slow down ectopic mineralization in Abcc6^−/−^ mice (Li et al., 2015; Pomozi et al., 2017). Finally, there have been two randomized clinical trials in PXE patients evaluating the effect of magnesium and the bisphosphonate etidronate respectively. Increased oral magnesium intake reduced skin elastic fiber calcification, albeit not statistically significant, and had no effect on ophthalmological outcomes (Rose et al., 2019). Oral etidronate therapy did affect progression of vascular mineralization but had no significant effects on eye symptoms (Kranenburg et al., 2018). While drug efficacy needs to be evaluated in patients, such trials are slow and require substantial amounts of resources (compound, time, labor). Moreover, toxicity and long-term effects are a major concern (Pazianas and Abrahamsen 2011; Moore et al., 2015).

Consequently, animal models seem to be a prerequisite for the discovery and testing of drugs that not only halt–but ideally also reverse–excessive calcification in PXE. Unfortunately, while multiple rodent models have been generated for PXE, mouse and rat models are not ideally suited for rapid testing of multiple drugs. Despite being more closely related to humans, ethical and economic drawbacks typically lead to smaller study cohort sizes. Moreover, the phenotypic effects of Abcc6 deficiency in these animals only become apparent after several weeks to months (Gorgels et al., 2005; Li et al., 2014), resulting in elongated exposure periods, high drug consumption and substantial husbandry costs.

Zebrafish models are much better suited for this endeavor. Husbandry is substantially different at lower costs per animal. Their high fecundity, small size and rapid *ex utero* development allow for larger cohorts that require less drug consumption with shorter exposure periods. Furthermore, gene and protein function largely overlaps with humans (Howe et al., 2013) and as zebrafish are maintained on an outbred background, they theoretically should better reflect the genetic diversity of patients than rodent models.

The ABCC6 gene has two orthologues–abcc6a and abcc6b–in zebrafish. While the function of abcc6b remains to be elucidated, abcc6a is a regulator of calcification (Mackay et al., 2015; Van Gils et al., 2018; Sun et al., 2021). We have previously reported on the development and phenotypic characterization of a CRISPR/Cas-9-mediated knockout zebrafish model (dubbed Cmg52) of the abcc6a gene. The Cmg52 allele is a four base-pair deletion (c.180delTCGG) in the second exon of abcc6a and predicted to result in p. R62Cfs*33 (Van Gils et al., 2018). Abcc6a loss-of-function results in progressive hypermineralization of axioskeletal bone structures in homozygous mutants but not in heterozygous animals, though the underlying mechanism remains unclear. In summary, excess mineral growth and fusion of vertebrae leads to a severe phenotype with spinal malformations and shorter stature in adult fish. Moreover, such hypermineralization is already apparent during larval development and semiquatifiable with knockout larvae having significantly more notochord sheath mineralization than their heterozygous and wild type siblings at 9–10 days post-fertilization (dpf) (Van Gils et al., 2018). This phenotype was confirmed to be similar to that of abcc6a missense mutants and recently another abcc6a knockout model (Mackay et al., 2015; Van Gils et al., 2018; Sun et al., 2021). A caveat to the use of zebrafish models for screening is that the expression site of abcc6a - which appears to coincide with osteoblast-like cells and, therefore, skeletal tissue - differs from that of ABCC6 in mammals (Mackay et al., 2015). For this reason we aimed to perform a proof-of-concept study using compounds reported in PXE literature. We hypothesized that, if treatment outcomes with these specific compounds are analogous for the mineralization phenotype of the abcc6a^cmg52/cmg52^ zebrafish and the soft tissue mineralizations of other models, the zebrafish model should be a valid tool in PXE research for drug screening purposes.

## 2 Materials and Methods

### 2.1 Zebrafish Husbandry and Phenotype

All animals were housed in the Zebrafish Facility of the Ghent Center for Medical Genetics on a 14 h light/10 h dark cycle utilizing semi-closed recirculating systems (ZebTEC, Tecniplast) kept at 27–28°C, pH 7.5 and conductivity ±500 µS. Adult and rearing zebrafish were fed twice a day, once with artemia (1579706, Ocean Nutrition) and once with dry food (GEMMA Micro 75-300, Skretting). Lines were maintained and outbred on an AB background. Animal experiments were approved by the Animal Experimentation Committee of the Ghent University and performed in accordance with the EU Directive 2010/63/EU for animal experiments.

For the compound screening studies, zebrafish allele cmg52 (https://www.zfin.org/ZDB-ALT-200727-1) was used and abcc6a^cmg52/+^ zebrafish (heterozygotes) were in-crossed to obtain abcc6a^cmg52/cmg52^ mutants (knockouts). As previously reported, in these homozygous abcc6a^cmg52/cmg52^ mutants abcc6a expression is severely diminished by the CRISPR/Cas9 induced c.180delTCGG allele variant (Van Gils et al., 2018). Consequently, abcc6a^cmg52/cmg52^ larvae more rapidly develop notochord mineralization with occasional vertebral fusion than carrier or wild type siblings. This phenotype can be analyzed as early as 10 days post-fertilization (dpf) by performing alizarin red S calcium deposit staining and semiquantifying notochord mineralizations.

### 2.2 Compound Screening Setup and Mineral Deposit Staining

Five compounds with previously reported anti-mineralizing activity were selected for a proof-of-concept approach. Treatment dosages were adopted from literature or based on in-house range-finding survivability testing on wild-type (WT) embryos (between 3-7 dpf; Supplementary Data S1–S3). Screening was performed using final doses of 80 µM vitamin K1 (95271, Sigma-Aldrich; Mackay et al., 2015), 20 µM sodium thiosulfate (72049, Sigma-Aldrich; toxicity test), 100 µM etidronate (P5248, Sigma-Aldrich; Apschner et al., 2014), 100 µM alendronate (PHR1599, Sigma-Aldrich; toxicity test) and 10 mM magnesium citrate (CDS000001, Sigma-Aldrich; toxicity test). VK1 was dissolved in 1:1 DMSO:ethanol (in accordance with Mackay et al., 2015), the other compounds were water-soluble. Larvae were not fed for the duration of the experiments as feeding introduces a variable influx of phosphate for mineralization and compounds might potentially bind to excess feed (Cotti et al., 2020).

Per experiment cmg52 heterozygotes were in-crossed using established protocols (Westerfield 2000) and resulting offspring was transferred per 100 into 90 mm petri dishes (F11093, M.L.S.) with E3-medium (5 mM NaCl, 0.17mM KCl, 0.33 mM CaCl_2_.2H_2_O, 0.33 mM MgSO_4_.7H_2_O, 5 mM HEPES, pH 7.4) + 0.00001% methylene blue (hence referred to as E3-medium). Medium was refreshed daily and only morphologically normal developing embryos were selected for further experiments at 3 dpf.

At 3 dpf embryos were dechorionated via pronase digestion if necessary and randomized into baskets in equal amounts (N ≤ 20 per basket; 734-0003, VWR) to allow quick swapping and minimal animal handling during the experiments. Baskets were then placed in 6-well plates filled either with 8 ml E3-medium + compound (i.e. treated) or E3-medium + carrier solution (i.e. controls [C]). Solutions were refreshed daily until 10 dpf at which point all larvae were euthanized (25x Tricaine for 10 min at room temperature) and fixated with 4% paraformaldehyde, 0.4 M PO_4_-buffer for 1 h at room temperature.

Larvae were then stained for mineral deposition using the Alizarin Red S (ARS) staining technique (Van Gils et al., 2018). Specimens were bleached for 30 min at room temperature with 1%H_2_O_2_, 1% KOH, 0.5% Triton X-100 and rinsed with distilled water to stop the reaction. Staining was performed overnight at 4°C using 0.05% ARS, 1% KOH, 0.5% Triton X-100 under gentle locomotion. Destaining was performed at room temperature with 30% glycerol and through incremental washing (30%, 50% 70 and 100% glycerol) specimens were stored in glycerol at 4°C. ARS-staining was performed concomitantly for control and compound treated groups of each experiment, sharing solutions and timing to minimize variance.

### 2.3 Image Analysis

Each group was examined and larvae with apparent spinal mineralization were transferred into Nunc™ glass bottom dishes (150680; Thermo Fischer Scientific) containing 100% glycerol and positioned for lateral view. Of these animals whole-body light microscopy images were taken with a Leica M165 FC microscope under identical conditions (e.g., zoom, aperture, light intensity) per control/compound duo. After imaging, all larvae were then transferred into 96-well plates at designated positions and stored at 4°C for genotyping. Larvae with notochord mineralization in regions other than the tip were considered to have “spinal mineralization”.

Images were processed using ImageJ (NIH, Bethesda, MD; Schneider et al., 2012) and spinal mineralization was semiquantified. Images were first converted into 16-bit (i.e., greyscale). tiff files. Excluding the notochord tip, all other segments of the notochord were then delineated and mineralized areas were segmented from the background using threshold analysis. The mineralized areas were then semiquantified per larva.

### 2.4 Abcc6a Genotyping

Ninety-six-well plates were batch-analyzed per experiment. Per well 100 µL 50 mM NaOH was added and plates were heated to 95°C for 20 min. Following neutralization of pH with 10 µL 1 M Tris-HCl, debris was pelleted and supernatant collected for genotyping.

Abcc6a primer sequences used in TD-PCR and cycle sequencing were: F: 5′-GGT​TTG​GAC​TGA​GCC​ATT​GT-3’; R: 5′-TCG​ACC​ACT​TTC​ACG​TTC​AC-3’. TD-PCR with 5 µL KAPA2G Robust Hotstart Ready Mix (KK5702, Sigma-Aldrich), 0.1 μL F-primer (30 µM), 0.1 μL R-Primer 30 µM), 2.8 µL ddH2O and 2 µL supernatant was performed (94°C, 4’ [94°C, 30”; 58 > 52°C, 30”; 72°C, 1’]x6; [94°C, 40”; 52°C, 40”; 72°C, 40”]x25; 72°C, 10′). Amplicons were purified with 1 µL of ExoAP mix (1 µL exonuclease I [M0293S, NEB], 4 µL antarctic phosphatase [M0298L, NEB], 15 µL ddH2O) to 5 µL PCR product and incubating the samples (37°C, 15’; 80°C, 20′).

Cycle sequencing (95°C, 5’ [95°C, 10”; 55°C, 5”; 60°C, 4’]x25) was then performed using the Big Dye™ Terminator v3.1 kit (4337457, Applied Biosystems). Products were purified via CleanDTR beads (CDTR-0050, GC Biotech) ethanol capturing and sequencing was performed by the Genome Sequencing Unit of the Ghent Center for Medical Genetics. Genotypes were manually checked with FinchTV software (Geospiza).

### 2.5 Statistical Analysis and Tables

Per experiment image data was stratified according to abcc6a genotype in SPSS26 software (IBM). Shapiro-Wilk normality testing was performed and extreme outliers, if present, were excluded. Effect of the compound on mineralization was determined by comparing control and treated groups with two-tailed *T*-tests or Mann-Whitney *U*-tests. Data was considered statistically significant at *p* < 0.05. Following relative normalization of data against the corresponding mean control value, tables (mean ± SD) were generated in Excel (Microsoft).

## 3 Results

### 3.1 Vitamin K1 Reduces Spinal Mineralization During Development

**FIGURE 1:**
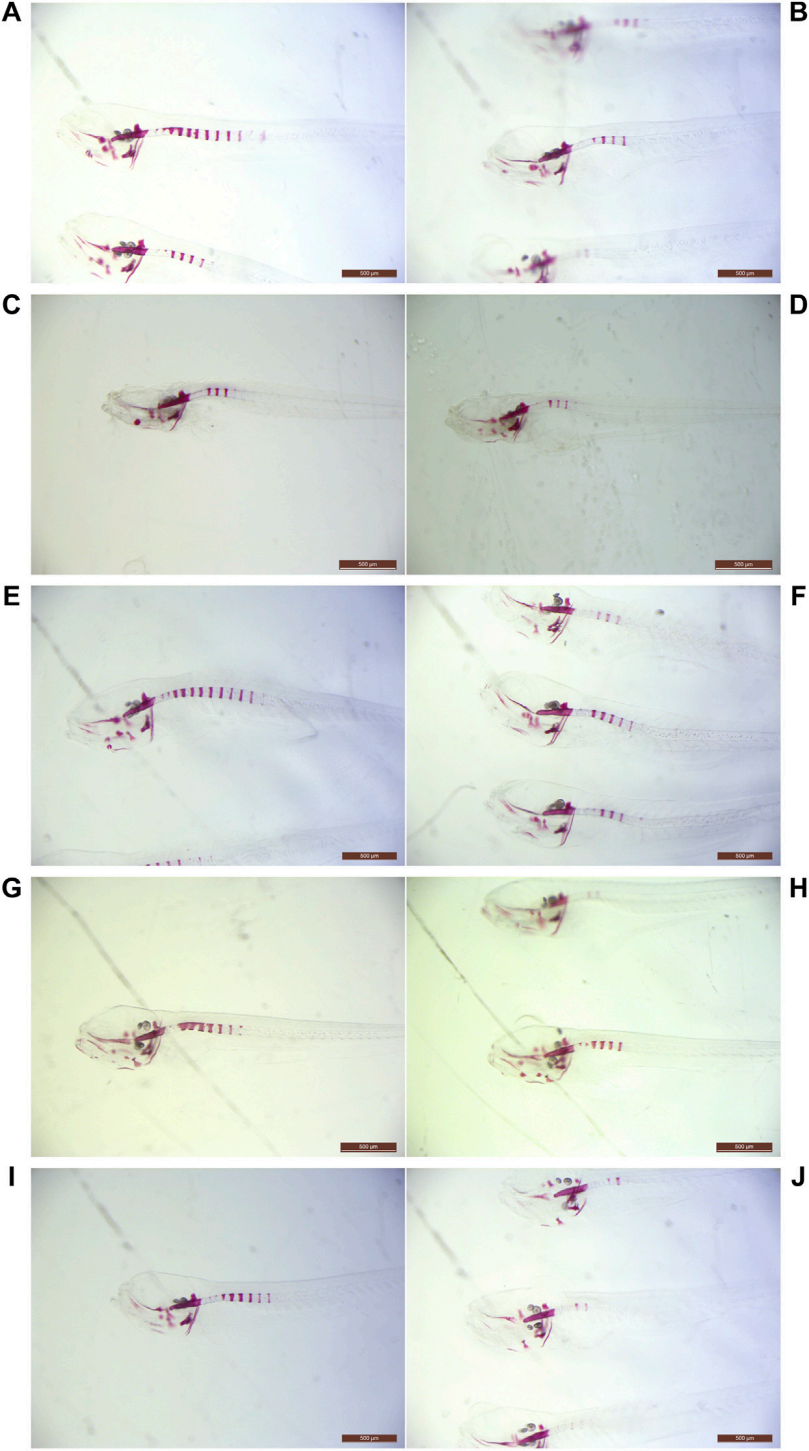
Light microscopy examples of ARS-stained abcc6a^cmg52/cmg52^ specimens. Examples of 10 dpf untreated **(** **A**,**C**,**E**,**G**,**I** **)** and treated **(** **B**,**D**,**F**,**H**,**J** **)** abcc6a^cmg52/cmg52^ animals. Compounds per set are **(** **A**,**B** **)** 80 µM vitamin K1, **(** **C**,**D** **)** 20 µM sodium thiosulfate **(** **E**,**F** **)** 100 µM etidronate, **(** **G**,**H** **)** 100 µM alendronate and **(** **I**,**J** **)** 10 mM magnesium citrate.

**FIGURE 2:**
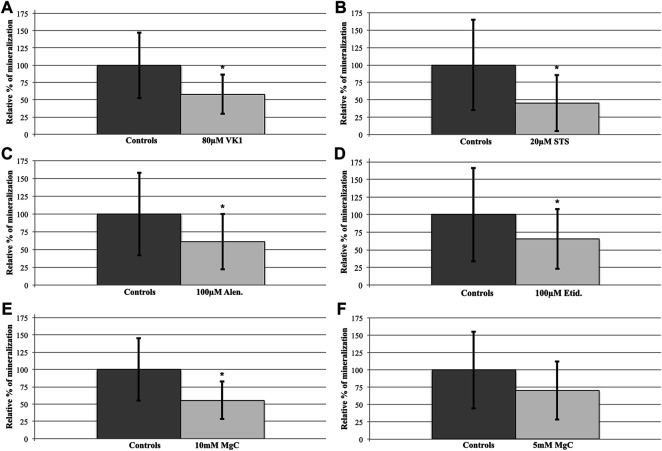
Compound treatment effects on the abcc6a^cmg52/cmg52^ spinal hypermineralization phenotype. After semiquantification and statistical analysis, mean ± SD percentile values of the spinal mineralization were normalized per compound to the respective control values of the untreated abcc6a^cmg52/cmg52^ fish. **(** **A** **)** 80 µM VK1 (58 ± 28) versus C (100 ± 48), 42% reduction **(** **B** **)** 20 µM STS (45 ± 40) versus C (100 ± 65), 55% reduction **(** **C** **)** 100 µM Etid. **(**65 ± 43) versus C (100 ± 67), 35% reduction **(** **D** **)** 100 µM Alen. (61 ± 39) versus C (100 ± 58), 39% reduction **(** **E** **)** 10 mM MgC (55 ± 27) versus C (100 ± 55), 45% reduction **(** **F** **)** 5 mM MgC (70 ± 42) versus C (100 ± 55), 30% reduction. Treatments resulted in significant reductions (*p < 0.05), except for 5 mM MgC.

One hundred twenty embryos were split into untreated (C: *n* = 60) and 80 µM VK1-treated (VK1: *n* = 60) cohorts at 3 dpf. During screening two VK1-treated larvae died, resulting in ARS-staining of 60 controls and 58 VK1-treated animals at 10 dpf. Upon genotyping only abcc6a^cmg52/cmg52^ larvae had developed mineralized segments other than the notochord tip (C: *n* = 15, VK1: *n* = 10; Table 1) with vitamin K1-treated knockouts showing a significant 42% reduction in mineralization compared to their control counterparts (*t*-Test: *p* < 0.05, Figures 1A,B, Figure 2A).

**TABLE 1:** Geno- and phenotypic distribution of the larvae analyzed at 10 dpf. Quantities and distribution of larvae per genotype and phenotype (i.e. presence of mineralized notochord sections other than the tip) are shown for each compound. WT = abcc6a^+/+^ wild types, Htz = abcc6a^cmg52/+^ heterozygotes, KO = abcc6a^cmg52/cmg52^ knockouts.

Cohorts	Spinal mineralization			No Spinal mineralization			Animal Total
	WT	Htz	KO	WT	Htz	KO	
Controls	0	0	15	14	28	3	60
80 µM VK1	0	0	10	11	36	1	58
Controls	0	0	20	28	54	11	113
20 µM STS	0	0	20	33	52	11	116
Controls	4	28	36	31	59	6	164
100 µM Etid	14	22	37	33	56	7	169
Controls	4	28	22	13	18	1	86
100 µM Alen	9	34	22	14	15	0	94
Controls	2	15	22	8	25	0	72
10 mM MgC	1	7	17	14	31	1	71
Controls	12	21	22	3	8	1	67
5 mM MgC	2	5	15	10	33	1	66

### 3.2 Sodium Thiosulfate Reduces Spinal Mineralization During Development, but Higher Doses Result in Ectopic Mineralization

**FIGURE 3:**
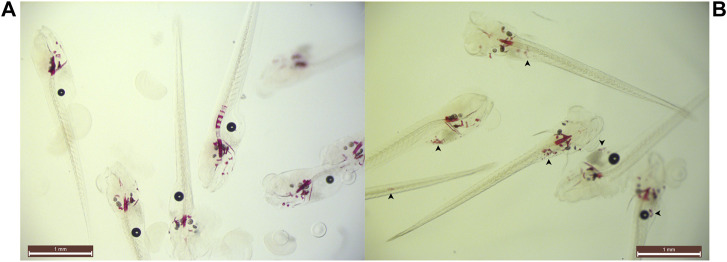
Example of ectopic speckling following 35 µM STS-treatment. Snapshot group images of **(** **A** **)** control and **(** **B** **)** 35 µM STS-treated larvae at 10 dpf with unknown genotypes. 35µM sodium thiosulfate abolished spinal mineralization and concomitantly ventral mineralized nodules were identified (arrowheads) in all treated animals. Opacity of the specimens prevented identification of the anatomical sites of the nodules.

STS survival testing indicated that embryos and larvae could be treated safely with 35 µM (Supplementary Data S1). However, 35 µM STS-treatment completely abolished spinal mineralization while simultaneously causing ventral mineral depositions in a speckled pattern for all treated animals (Figure 3). Therefore we reduced the dosage until this pattern disappeared at 20 µM sodium thiosulfate. A total of 120 untreated and 120 20 µM STS-treated embryos were processed in 2 experiments. During treatment, 7 untreated and 4 STS-treated larvae expired (Table 1). Following genotyping, only abcc6a knockouts had spinal hypermineralization (C: *n* = 20; STS: *n* = 20). On average, spinal mineralization was significantly reduced by at least 55% following sodium thiosulfate treatment (Mann-Whitney *U*-Test: *p* < 0.05 [C: *n* = 10, STS: *n* = 8, 55%] and *p* < 0.05 [C: *n* = 10, STS: *n* = 12, 57%], Figures 1C,D, Figure 2B).

### 3.3 Bisphosphonate treatment Significantly Reduces Spinal Mineralization in abcc6a^cmg52/cmg52^ Larvae

To evaluate the effect of etidronate (etid.) on the spinal mineralization phenotype, 175 embryos were treated with 100 µM etidronate and compared to 175 untreated larvae in two experiments. As 5 untreated and 2 etid.-treated larvae died and genotyping failed for 6 and 4 larvae respectively, 164 untreated and 169 etid.-treated larvae were analyzed (Table 1). Spinal mineralization was found for all genotypes, but was only excessive in the knockouts as expected. The number of WT animals with spinal mineralization was too low (*n* < 5 per group) to assess an effect of etidronate on the spinal mineralization. For heterozygotes one experiment had sufficient quantities to perform the analysis, but etidronate treatment had no significant effect (Mann-Whitney *U*-Test: *p* > 0.05 [C: *n* = 28, Etid: *n* = 21]). By contrast, mean mineralization in abcc6a knockouts was significantly reduced by approximately 35% following etidronate treatment (*t*-Test: *p* < 0.05 [C: *n* = 14, Etid: *n* = 9] and Mann-Whitney *U*-Test: *p* < 0.05 [C: *n* = 22, Etid: *n* = 28], Figures 1E,F, Figure 2C).

Alendronate was tolerated at similar doses as etidronate for the duration of the experiment. We treated 100 larvae with 100 µM alendronate and compared them to 100 controls. Respectively 6 controls and 4 treated animals expired and genotyping failed for 8 and 2 animals resulting in analysis of 86 control and 94 alen.-treated larvae (Table 1). Again, mineralization was detected for all genotypes, but without sufficient numbers of WT. In heterozygotes mineralization was not significantly affected by alendronate treatment (Mann-Whitney *U*-Test: *p* > 0.05 [C: *n* = 28, Alen: *n* = 34]). However, treatment significantly reduced spinal hypermineralization by approximately 39% in abcc6a knockouts (Mann-Whitney *U*-Test: *p* < 0.05 [C: *n* = 22, Alen: *n* = 22], Figures 1G,H, Figure 2D).

### 3.4 Magnesium citrate Significantly Affects Spinal Mineralization in abcc6a^cmg52/cmg52^ Larvae

Initially, an experiment with 10 mM MgC-treatment doses (C: *n* = 80, MgC: *n* = 80) was performed and 8 untreated and 9 MgC-treated animals perished resulting in comparison of 72 C and 71 MgC larvae (Table 1). Treated abcc6a knockouts had a significant decrease in spinal mineralization by 45% (*t*-Test: *p* < 0.05 [C: *n* = 22, MgC: *n* = 17], Figures 1I,J, Figure 2E). Similar to the bisphosphonate treatments, some WT (C: *n* = 2, MgC: *n* = 1, not analyzed) and heterozygous larvae also had mineralized spinal sections. Magnesium citrate treatment significantly reduced mineralization in heterozygotes by 77% (Mann-Whitney *U*-Test: *p* < 0.05 [C: *n* = 15, MgC: *n* = 7]).

In order to determine a minimal effective dose we performed an additional experiment with 5 mM MgC as a therapeutic dose (C: *n* = 80, MgC: *n* = 80). We analyzed 79 untreated and 67 MgC-treated larvae as respectively 1 and 9 animals perished and sequencing of 4 MgC-treated animals had failed (Table 1). Again, spinal mineralization was detected in all genotypes but analysis of WT was not feasible due to the low number of animals. In this setup, 5 mM MgC-treatment did not significantly affect mineralization in heterozygous larvae (18% reduction, Mann-Whitney *U*-Test: *p* > 0.05 [C: *n* = 21, MgC: *n* = 5]). Similarly, spinal mineralization in knockout animals was non-significantly reduced (30%, Mann-Whitney *U*-Test: *p* > 0.05 [C: *n* = 22, MgC: *n* = 14], Figure 2F).

## 4 Discussion

To date the lack of curative treatment options for PXE remains one of the main limitations in patient management. One avenue for (novel) drug discovery is to perform compound screening on animal models with a well-characterized phenotype. Therefore, we evaluated our zebrafish abcc6a knockout line, carrying the Cmg52 allele, as a candidate model. We hypothesized that treatment with compounds implicated in PXE (VK1, STS, bisphosphonates and magnesium) would have a similar effect on the spinal hypermineralization phenotype as what has been reported in PXE murine models and/or patients.

### 4.1 Vitamin K1

**FIGURE 4:**
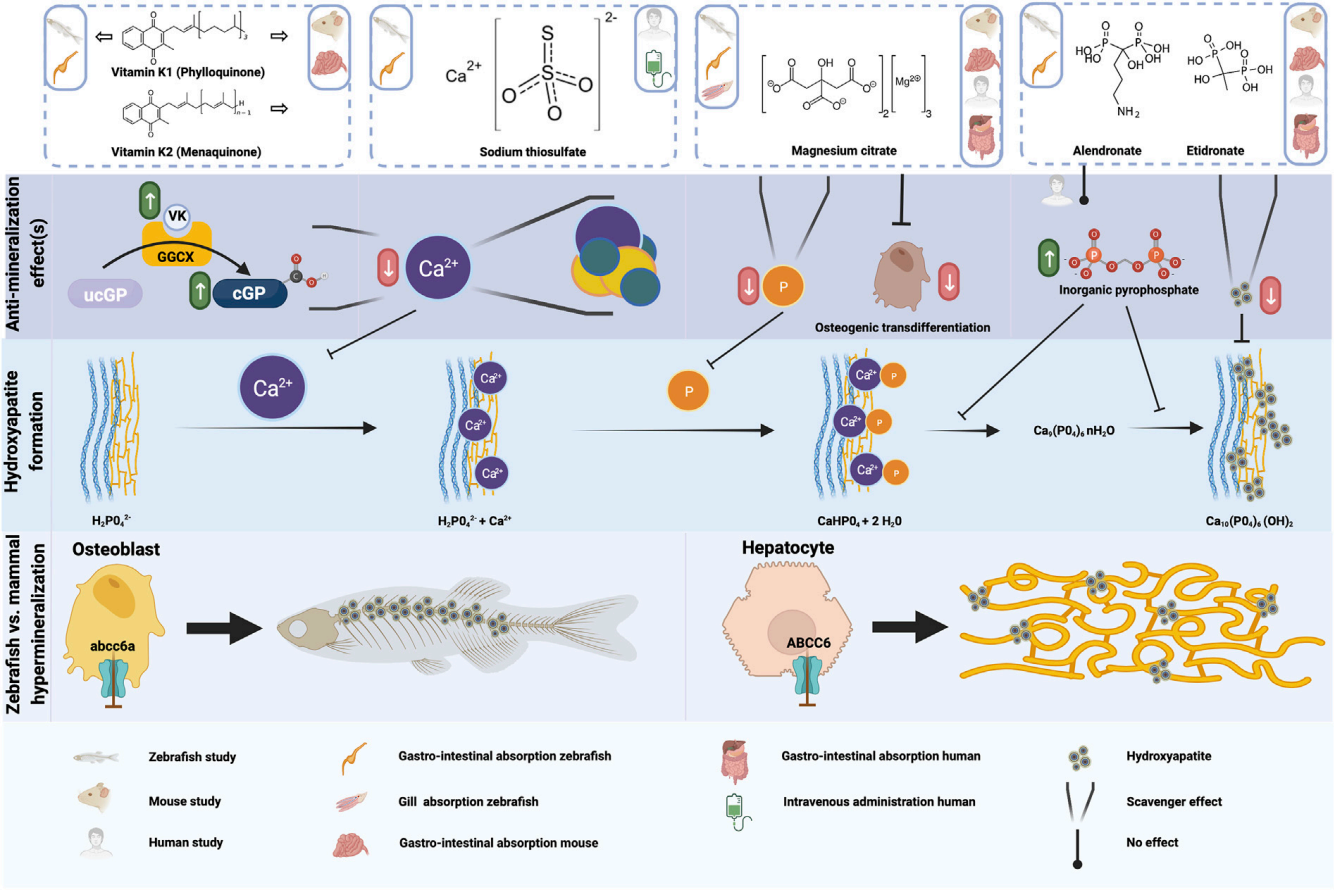
Schematic representation of the anti-mineralization effects of the tested compounds. For the compounds evaluated in the present study, the type of administration or absorption is mentioned in the top row, as well as the type of administration used in any mouse or human studies which were done with this compound. The anti mineralizing effects of the different compounds are shown, as well as where these effect take place in the cascade to form hydroxyapatite crystals. Finally, the differences between the hypermineralization in zebrafish and mammals with respect to abcc6a and ABCC6 deficiency are shown. Figure created with BioRender.com.

Mackay et al. and Sun et al. reported a significant reduction (rough estimate ±30–45%) in spinal mineralized area when their zebrafish abcc6a^−/−^ models were treated with 80 µM vitamin K1 (Mackay et al., 2015; Sun et al., 2021). In our setup we found a comparable reduction of 42%, verifying that VK1-treatment has an effect on spinal mineralization, at least in abcc6a^−/−^ zebrafish models. While reduced VK1 serum levels have been reported in PXE patients (Vanakker et al., 2010), beneficial effects of VK1 supplementation against ectopic mineralization could not be demonstrated in rodent models (Brampton et al., 2011; Gorgels et al., 2011; Jiang et al., 2011). This was also the reason behind the lack of human trials for vitamin K supplementation. Expression sites of abcc6a in zebrafish and ABCC6 in mammals–as well as parts of the affected mineralization sites (Mackay et al., 2015; Sun et al., 2021)–are considerably different which could explain the differential effect of VK1. ABCC6 is predominantly expressed by hepatic (and renal) tissue in mammals with loss-of-function primarily resulting in ectopic calcifications in skin, eye and vascular tissues (Gorgels et al., 2005). By contrast, abcc6a expression coincides with osteoblast-like cells in the craniofacial bones and notochord and, concomitant with VK homeostasis markers, appears to be enriched in the intervertebral disc regions (Fernández et al., 2015; Mackay et al., 2015) (Figure 4). Hence, Abcc6a expression is more local at the sites of hypermineralization compared to ABCC6. Though no conclusive data has been established to date, VK might mitigate abcc6a-related dysfunction (e.g. oxidative stress, VK-dependent carboxylation) more readily at the local level in zebrafish compared to the peripheral effects in mammals (Fernández et al., 2015; Nollet et al., 2019). Another consideration herein could be the difference in administration of the compounds. While mouse models were “periodically” subjected to VK supplementation via the chow or via peritoneal injection, zebrafish models were continuously exposed due to immersion into the compound solution. Moreover, zebrafish were treated much earlier during development (i.e. starting in the embryonic stage and pre-mineralization) while only one study investigated the effect of vitamin K treatment in developing mice (Brampton et al., 2011).

While rodent model data appear negative, the existence of a PXE-like disorder with coagulation factor deficiency–caused by GGCX (Gamma-Glutamyl Carboxylase) mutations–where VK-dependent carboxylation is (nearly) abolished suggests an important role for VK in mineralization homeostasis (Vanakker et al., 2010). In addition, warfarin treatment–which blocks vitamin K recycling–exacerbates the aberrant mineralization in both murine and zebrafish PXE models (Li et al., 2013; Mackay et al., 2015). Taken together, there might be a role for VK in mammalian PXE-related mineralization, perhaps as an attenuator of processes via matrix gla protein (MGP) (Theuwissen et al., 2012). Moreover, VK may have a slow impact as beneficial effects have only been reported in a single human cardiovascular disease clinical trial after 3 years of vitamin K supplementation (Vlasschaert et al., 2020). Considering any trial investigating VK in PXE murine models has not lasted longer than a few months, such putative late-onset effects could have been missed.

### 4.2 Sodium Thiosulfate

Sodium thiosulfate functions as an antioxidant agent and as a chelator capable of dissolving precipitated calcium phosphate-based crystals (Figure 4). As such it has been used to treat various ectopic mineralization disorders (e.g. calciphylaxis in chronic kidney disease (Peng et al., 2018), calcinosis cutis (Von Hodenberg et al., 2020; López-Sundh et al., 2021) and one case of severe pseudoxanthoma elasticum (Omarjee et al., 2020)). In this PXE patient, many calcified lesions ameliorated and calcific stenosis of celiac and mesenterial arteries was resolved following treatment (Omarjee et al., 2020). Unfortunately, the therapy requires careful follow-up and lesions quickly reformed after cessation of treatment (Omarjee et al., 2020).

Here we report that 20 µM STS-treatment significantly reduced the spinal mineralization in abcc6a^cmg52/cmg52^ zebrafish by 55%. It appears that dosage should be carefully considered as, despite its tolerability, we discovered a peculiar side effect (i.e. the ventral ectopic spots in wild type, heterozygous and knockout fish) of 35 µM STS-treatment. We are unsure how this spotting pattern occurred as STS-chelated calcium and phosphate particles would normally be excreted into the environment. These ventral sites appear to rudimentary co-locate with the gastrointestinal tract (GIT) but further investigation is required to understand the underlying mechanisms. We see no explanation for this from our experimental set-up; the zebrafish GIT has pH-levels ≥7.5 and calcium thiosulfates would remain highly soluble. Moreover, the medium was refreshed daily, thereby removing excreted particles regularly. To our knowledge, aberrant calcifications due to STS have not been reported in literature. Sodium thiosulfate appears to be generally tolerable though some side effects have been reported with intravenous administration causing nausea, vomiting and metabolic acidosis (Peng et al., 2018; Omarjee et al., 2020) and topical STS-treatment occasionally causing skin irritation (Ma et al., 2019). Thus, sodium thiosulfate might be a promising candidate drug but optimized treatment schedules and diligent patient follow-up appear warranted to minimize side effects and impact on quality of life.

### 4.3 Bisphosphonates

Bisphosphonates are a group of compounds with structural analogy to inorganic pyrophosphate, an inhibitor of calcification (Kuźnik et al., 2020). Contrary to inorganic pyrophosphate, bisphosphonates are chemically stable and have dual activity. On the one hand these compounds bind calcium phosphate particles thereby preventing growth and sedimentation (Figure 4). On the other hand they inhibit bone resorption activity by inducing apoptosis of osteoclasts (Hughes et al., 1995; Rodan and Fleisch 1996). The mechanism by which bisphosphonates exert their anti-osteoclast activity is dependent on their chemical structure. Simple or non-nitrogen-based compounds (e.g. etidronate) form toxic adenosine triphosphate analogs once resorbed from bone by osteoclasts, while nitrogen-containing compounds (e.g. alendronate) inhibit farnesyl diphosphate synthase activity, ultimately suppressing resorption and inducing apoptosis (Rezska and Rodan 2003). Additionally, the nitrogen-based bisphosphonates bind to bone mineral with higher affinity than their simpler counterparts (Kuźnik et al., 2020).

Etidronate and alendronate have been evaluated prior in relation to PXE (and GACI). Intraperitoneal injections of etidronate prior to cardiac injury prevented the dystrophic cardiac calcification phenotype reported in Abcc6^−/−^ mice (Pomozi et al., 2017). Similarly, subcutaneous injection or high doses of etidronate in feed (equivalent to 12x daily dose to treat osteoporosis in humans) halted ectopic mineralization in the muzzle of Abcc6^−/−^ mice (Li et al., 2015, Li et al., 2016). Most importantly, a clinical trial found etidronate therapy halts arterial calcification in most vascular beds–apart from cardiac arteries–without affecting choroidal neovascularization in PXE (Kranenburg et al., 2018; Bartstra et al., 2020; Risseeuw et al., 2020). In addition, etidronate-coated nanoparticles designed to target elastin resolved mineralization deposits in Enpp1^−/−^ rat aortic cultures (Keuth et al., 2020). Finally, treatment of enpp1^−/−^ zebrafish with 100 µM etidronate significantly reduced spinal hypermineralization during embryonic/larval development (Apschner et al., 2014). In agreement with the literature, treatment of our abcc6a knockout zebrafish, but not wild type or heterozygotes, with 100 µM etidronate during development significantly reduced (35%) spinal hypermineralization, likely by calcification inhibition akin to pyrophosphate. It also confirms that etidronate treatment can be effective in patients with generalized arterial calcification of infancy due to biallelic ABCC6 mutations (Nitschke et al., 2012).

In contrast to earlier reports in mice, we also found alendronate treatment to significantly reduce mineralization (Li et al., 2015; Pomozi et al., 2017). Besides putative species-dependent differences, this can also be explained by the experimental setup. Similar to etidronate, exposure to alendronate occurs much earlier in our model (i.e. prior to notochord mineralization) and was continuous instead of periodic (swimming medium versus dietary intake/injection). Nonetheless, while alendronate is typically administered to patients at much lower dosages than etidronate, our alendronate dose would equate to roughly a 20x higher dosing, which is also much higher than the 12x higher dose applied to mice (Li et al., 2015). This could be due to the higher affinity for bone of nitrogen-based bisphosphonates, suggesting that alendronate is not a feasible therapeutic option for pseudoxanthoma elasticum patients.

### 4.4 Magnesium Citrate

Magnesium has frequently been implicated as a modulator of cardiovascular disease risk, acting as both a phosphate-binding competitor of calcium - thus delaying calcium phosphate crystal growth - and a suppressor of osteogenic transdifferentiation (Ter Braake et al., 2017) (Figure 4). Similarly, dietary magnesium oxide supplementation in Mendelian soft connective tissue mineralopathies inferred beneficial effects. In PXE mouse models a 4–5x increased intake slowed ectopic calcification formation and growth (LaRusso et al., 2009; Gorgels et al., 2010; Kupetsky et al., 2013) while reduced intake exacerbated the phenotype (Jiang and Uitto 2012). Moreover, one study hinted that longer-term dietary magnesium intake might reduce carotid intima-media thickness scores in Abcc6^−/−^ mice, suggesting elevated magnesium intake can ameliorate vascular disease (Kupetsky-Rincon et al., 2012). Additionally, *in utero* development of GACI was prevented if pregnant mice were placed on a high magnesium diet (Kingman et al., 2017). A clinical trial in pseudoxanthoma elasticum patients, however, failed to demonstrate a clear effect of magnesium, though the data interpretation was hampered by clinical variability between patients, confounding effects of parallel treatments (e.g. for the ophthalmological endpoints) and very strict limitations on magnesium dosage (despite it being well tolerated) (Rose et al., 2019). While promising, early and prolonged exposure to high doses (i.e. 10x increase) of dietary magnesium may negatively affect bone mineralization (Ter Braake et al., 2020).

In our experiments we used magnesium citrate. Citrate is suggested to have a regulatory role in bone matrix as it binds to hydroxyapatite crystals and even inhibits transformation of precursor molecules into hydroxyapatite crystals (Hu et al., 2010; Wu et al., 2021). Citrate had not been linked to ectopic mineralization until recently when ANKH (progressive ankylosis protein homolog) was reported to transport both citrate and ATP (Szeri et al., 2020). As ANKH is part of the ectopic mineralization paradigm and appears to be downregulated in PXE fibroblasts (Boraldi et al., 2014; Van Gils et al., 2019) citrate supplementation could potentially benefit pseudoxanthoma elasticum patients. We showed that magnesium citrate has a dosage dependent beneficial effect on the spinal hypermineralization phenotype in the abcc6a^cmg52/cmg52^ larvae, reaffirming the existing literature on magnesium. The extent of the contribution of citrate to the observed anti-mineralization effect remains to be determined, but magnesium and possibly citrate appear to be interesting candidates for therapeutic intervention in pseudoxanthoma elasticum.

In conclusion, our data demonstrate the utility of our CRISPR/Cas9 abcc6a knockout zebrafish model for PXE-specific compound screening studies. While the spinal phenotype certainly differs from the mammalian and patient phenotype, the PXE zebrafish model has several significant benefits. The outbred background paired with high fecundity (i.e. increased sample sizes) would likely enhance the robustness of identified correlations as it better mimics the diversity in genetic background of patients. In addition, the relatively speedy analysis and reduced amounts of compound necessary assert zebrafish as an interesting first line model for discovery of therapeutics or for identification of repurposable drugs already approved for patient care (Brueggeman et al., 2019; Widrick et al., 2019).

## Data Availability

The original contributions presented in the study are included in the article/Supplementary Material, further inquiries can be directed to the corresponding author.

## Abbreviations

Abcc6(a), ATP-binding cassette, sub-family C, member 6(a); Alen, alendronate; ARS, alizarin red S; C, controls; CRISPR, clustered regularly interspaced short palindromic repeats; Dpf, days post-fertilization; ENPP1, ectonucleotide pyrophosphatase/phosphodiesterase 1; Etid, etidronate; MgC, magnesium citrate; PXE, pseudoxanthoma elasticum; STS, sodium thiosulfate; VK, vitamin K; WT, wild type.
