# Supplementary material for: The Abcc6a Knockout Zebrafish Model as a Novel Tool for Drug Screening for Pseudoxanthoma Elasticum
Source: Front Pharmacol. 2022 Mar 4;13:822143. doi: 10.3389/fphar.2022.822143 (PMC8934400; doi:10.3389/fphar.2022.822143)
Supplement: Supplementary file 1 [file Table1.DOCX]

**SUPPLEMENTAL DATA**

***Table S1. Survivability of embryonic and larval exposure to sodium thiosulfate***

Survivability screening was performed for STS from 3 to 7 days post-fertilization using wild type animals. Animals were morphologically checked and relative survivability is shown in brackets. Dosages up to 40µM STS appear to be tolerable.

| ***Abcc6a^+/+^*** | **Morphologically healthy larvae (N) during sodium thiosulfate exposure 3-7 dpf** | | | | |
| --- | --- | --- | --- | --- | --- |
|  | **0µM (Controls)** | **20µM** | **30µM** | **40µM** | **50µM** |
| **3 dpf** | 80 (100%) | 80 (100%) | 80 (100%) | 80 (100%) | 80 (100%) |
| **4 dpf** | 78 (97.5%) | 80 (100%) | 80 (100%) | 80 (100%) | 77 (96.3%) |
| **5 dpf** | 78 (97.5%) | 79 (98.8%) | 80 (100%) | 80 (100%) | 74 (92.5%) |
| **6 dpf** | 78 (97.5%) | 78 (97.5%) | 79 (98.8%) | 78 (97.5%) | 72 (90.0%) |
| **7 dpf** | 77 (96.3%) | 78 (97.5%) | 77 (96.3%) | 78 (97.5%) | 69 (86.3%) |

***Table S2. Survivability of embryonic and larval exposure to alendronate***

Survivability screening was performed for alendronate from 3 to 7 days post-fertilization using wild type animals. As etidronate was considered effective at 100µM we opted to perform testing in this range. Animals were morphologically checked and relative survivability is shown in brackets. Dosage appeared tolerable for all conditions tested.

| ***Abcc6a^+/+^*** | **Morphologically healthy larvae (N) during alendronate exposure 3-7 dpf** | | | |
| --- | --- | --- | --- | --- |
|  | **0µM (Controls)** | **80µM** | **100µM** | **120µM** |
| **3 dpf** | 48 (100%) | 50 (100%) | 50 (100%) | 50 (100%) |
| **4 dpf** | 48 (100%) | 50 (100%) | 50 (100%) | 50 (100%) |
| **5 dpf** | 48 (100%) | 50 (100%) | 50 (100%) | 50 (100%) |
| **6 dpf** | 48 (100%) | 49 (98.0%) | 50 (100%) | 49 (98.0%) |
| **7 dpf** | 47 (97.9%) | 49 (98.0%) | 49 (98.0%) | 48 (96.0%) |

***Table S3. Survivability of embryonic and larval exposure to magnesium citrate***

Wild type animals were immersed in various concentrations of magnesium citrate for 3-7 dpf and survivability was checked for the duration. Counted animals appeared morphologically normal. Relative survivability is shown in brackets. Dosages up to 10mM magnesium citrate were tolerated but more larvae expired starting 20mM with 30mM appearing to be toxic.

| ***Abcc6a^+/+^*** | **Morphologically healthy larvae (N) during magnesium citrate exposure 3-7 dpf** | | | | |
| --- | --- | --- | --- | --- | --- |
|  | **0mM (Controls)** | **5mM** | **10mM** | **20mM** | **30mM** |
| **3 dpf** | 40 (100%) | 40 (100%) | 40 (100%) | 40 (100%) | 40 (100%) |
| **4 dpf** | 40 (100%) | 40 (100%) | 40 (100%) | 40 (100%) | 38 (95.0%) |
| **5 dpf** | 40 (100%) | 40 (100%) | 40 (100%) | 39 (97.5%) | 35 (87.5%) |
| **6 dpf** | 40 (100%) | 40 (100%) | 40 (100%) | 39 (97.5%) | 30 (75.0%) |
| **7 dpf** | 40 (100%) | 39 (97.5%) | 40 (100%) | 37 (92.5%) | 23 (57.5%) |
